# Grain-size distribution dataset of lagoonal and riverine coastal placer deposits along the southeastern coast of Sri Lanka

**DOI:** 10.1016/j.dib.2024.110726

**Published:** 2024-07-06

**Authors:** Chaturanga Sandaruwan, Nadeesha Madugalla, Madurya Adikaram, Amarasooriya Pitawala

**Affiliations:** a Postgraduate Institute of Science, University of Peradeniya, Peradeniya, 20400, Sri Lanka; b Department of Physical Sciences, Faculty of Applied Sciences, South Eastern University, Sammanthurai, 32200, Sri Lanka

**Keywords:** Garnet placers, Gradistat, Ilmenite placers, Swash sediments, Tri-variate plot

## Abstract

The dataset contains grain size data of placer and non-placer sediments in lagoonal and riverine beaches of southeastern part of Sri Lanka. A total of 124 swash sediment samples were collected from a 70 km long coastline with an interval of 500 m. Placer sediments in the area mainly have mineralogy of ilmenite, zircon and almandine while non-placers are quartz, albite and calcite. After dry sieving, the grain size distribution (GSD) analyses were carried out on each sample using the Gradistat Excel template. Placer deposits result coarse-skewed leptokurtic to platykurtic fine sand distributions while non-placers are medium sand-grained. The dataset can be used to interpret the deposition environment and transportation dynamics. Further, they can be used to study the southwestern coastline of the Bay of Bengal, juvenile crust sediments of Grenvillian age, alongshore and fluvial sediment dynamics, depositional and erosion processes, geohazards assessments and heavy mineral deposits.

Specifications Table

**Table:** 

Subject	Earth-Surface Processes
Specific subject area	Variations of grain size statical parameters between red, black placers and siliciclastic deposits; longshore, lagoonal and riverine deposit
Type of data	Table, Graph, Figure Raw, Analyzed, Filtered
Data collection	From October 2019 to February 2020, 124 sediment samples were collected from the middle of the swash zone to depths of 15–30 cm. Here, a plastic shovel and zip lock bags and a 0.5 km station interval were used. In there, samples were occupied from black, red placers, and siliciclastic deposits. GPS coordinates were recorded, and samples were taken to a laboratory. The sample preparation involved processing 100 g of sediment fractions using the coning and quartering method, removing carbonate and salt coatings (10 % HCl and deionized water), and drying in an oven at 60 °C for 24 h. The sieving was performed using 1 ϕ interval ASTM sieves, Retsch vibratory sieve shaker (AS 200 digit), and laboratory analytical balance for 15 min. The grain size distribution parameters, their graphical analysis and mapping were evaluated using the Gradistat Excel template, Origin Pro (2021) and Arc GIS (10.8).
Data source location	Institution: Faculty of Applied Sciences, South Eastern University, Sammanthurai, 32,200, Sri Lanka Region: South Eastern coast of Sri Lanka Country: Sri Lanka Latitude and longitude for collected sample: 7° 16′ 16.1′′ N – 81° 52′ 03.5′′ E and 6° 44′ 49.1′′ N – 81° 48′ 37.5′′ E
Data accessibility	Repository name: Mendeley Data Data identification number: DOI: 10.17632/vzpxszhc8x.1 Direct URL to data: https://data.mendeley.com/datasets/vzpxszhc8x/1
Related research article	C. Sandaruwan, N. Madugalla, M. Adikaram, A. Pitawala, and T. Udagedara, “Microtexture and grain size characteristics of lagoonal and riverine coastal deposits along the southeastern coast of Sri Lanka: implication for paleoenvironment,” *Arab. J. Geosci.*, vol. 16, no. 2, 2023, doi: 10.1007/s12517-022-11149-4. [1]

## 1 Value of the Data

• The data pertains to unexplored heavy mineral placers in the southeastern coast of Sri Lanka.
• The data focuses on the southwestern coast of the Bay of Bengal and examines variations in grain size distribution (GSD) [2].
• Vijayan Complex, which is the basement and sediment source of study area, is related to the evolution of an island arc, juvenile crust of Grenvillian age, Lützow-Holm Bay region in East Antarctica [3,4].
• The data show the GSD variations between the following: ilmenite, garnet placer deposits and siliciclastic sediment; crescent-shaped bays and straight beach sediment; numerous fluvial outlets and their gradual sinking distance increment on beach; and alongshore (∼70 km) beach sediments.
• The data can be reused for the studies of alongshore and fluvial sediment dynamics, depositional and erosion processes, engineering applications, Precambrian host rock, renewable heavy minerals and Bengal Bay sediment.
• The data can be beneficial to heavy mineral industries, sedimentologists, researchers, and civil engineers.

## 2 Background

The dataset was designed to distinguish grain size characteristics, sediment transport and depositional processes, and depositional environments of heavy placers from siliciclastic deposits. Also, the dataset can be used to understand the alongshore and fluvial sediment dynamics, nearshore coastal processes, coastal erosion, temporal variations, geohazards assessments and Bengal Bay sediments. This article presents raw data that are not included in the related research article. The raw dataset may be useful to researchers for creating cumulative passing presentation graphs and various bivariate graphs and statical analyses.

## 3 Data Description

**Fig. 1:**
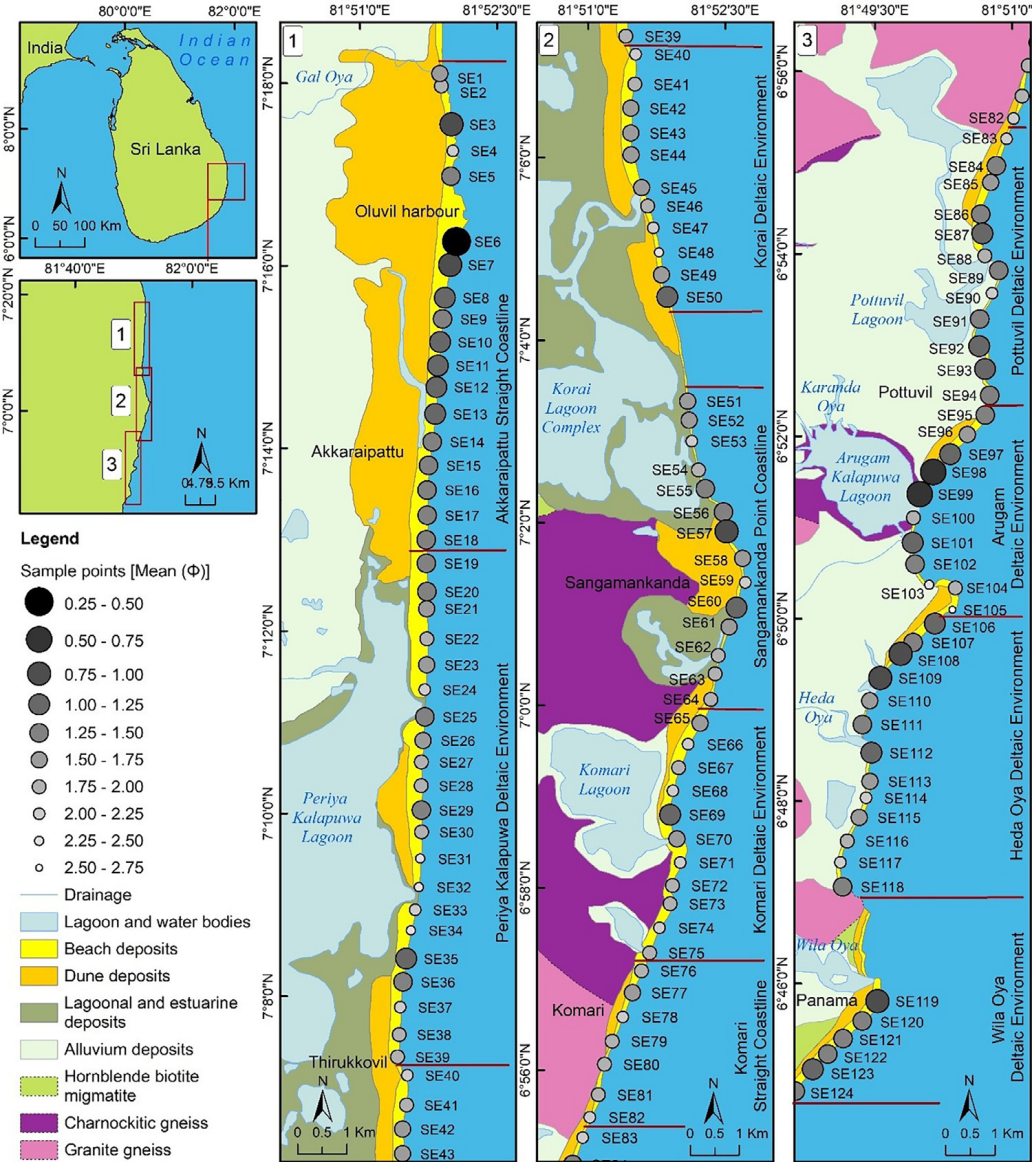
Geological map of the study area shows the sampling locations, lagoonal and riverine environments.

**Fig. 2:**
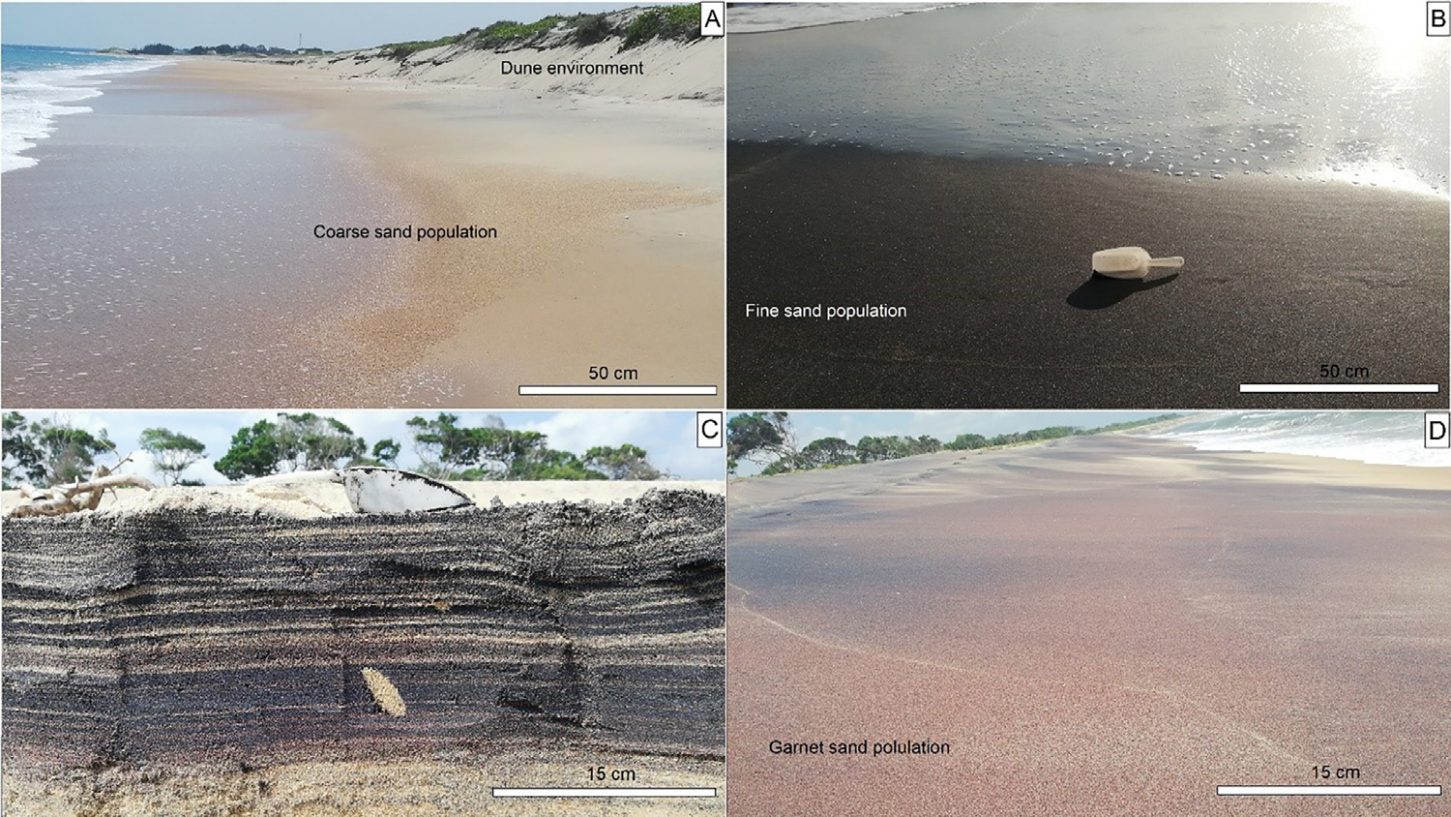
Examples of the sedimentary deposits from which the samples were taken. A) Siliciclastic deposits, B) and C) ilmenite placers and their vertical profile, and D) Garnet placers. The scale of plastic shovel is nearly 15 cm long.

In Fig. 1, symbols of the sample points are expressed as the mean values. Ilmenite placers of the area are denoted by the mean values > 2, while the Heda Oya deltaic environment shows the garnet deposits. Most of the placers can be found near fluvial outlets. The mean values of Akkaraipattu beach samples show an increasing trend towards the south direction. Fig. 2 shows the field photographs of the different deposits found in the study area.

The main fluvial outlets of Gal Oya, Karanda Oya, Heda Oya and Wil Oya result in between SE1, SE99-SE100, SE111-SE112 and SE118-SE119, respectively. Mostly, scatter distribution of fine sand placers can be observed around the southern part of these fluvial outlets (Fig. 1). The heavy mineral contents of Komari and Periya Kalapuwa are observed to be higher black in color (Fig 2B-C). Whereas, the beach sector in between Heda Oya and Wila Oya shows a high amount of garnets by giving the red color nature to the beach (Fig. 2D). The mineralogy of the area's placer deposits consists primarily of ilmenite, zircon, and almandine, while non-placers include quartz, albite, and calcite [1].

As geomorphological observations, the northward development of spits with granitic gneiss rock boulders resulted along the nearshore areas of Sangamankanda to Panama beach. Further, the southern half of the present study area showed a higher number of coastal dunes. Retention of heavy mineral populations is observed to be on the berm zone or swash zone with a depth of ∼10–15 cm (Table 1).

**Table 1:** Location data and grain size parameters of the samples in phi scale. The underlined and bolded mean values show the placers of the area.

S. ID	Latitude	Longitude		Mean (Φ)	Sorting (Φ)	Skewness (Φ)	Kurtosis (Φ)
SE1	7.30178	81.864562	Akkaraipattu Straight Coastline	1.6640	0.7580	0.0608	1.1634
SE2	7.299512	81.864851		1.8748	0.7417	0.0081	0.8911
SE3	7.292519	81.866721		0.7736	0.8530	0.0955	1.1142
SE4	7.287708	81.866951		**2.0399**	0.6528	−0.0183	0.7383
SE5	7.283046	81.866593		1.3075	0.8381	−0.0251	1.0028
SE6	7.271151	81.867633		0.4739	1.0773	0.2234	0.9461
SE7	7.266865	81.866478		0.9713	0.7932	0.2432	0.9015
SE8	7.260875	81.865497		1.2441	0.7688	−0.0404	1.0261
SE9	7.257002	81.865063		1.3106	0.7274	−0.0681	1.1949
SE10	7.252781	81.864597		1.2118	0.7474	−0.0374	0.9988
SE11	7.248464	81.864219		1.2274	0.7382	−0.0693	1.0341
SE12	7.244557	81.86393		1.0688	0.8270	−0.0107	0.9626
SE13	7.23965	81.863739		1.1044	0.8014	−0.0654	0.9588
SE14	7.234558	81.863181		1.2738	0.7053	−0.0929	1.1346
SE15	7.230248	81.862507		1.3409	0.7163	−0.0838	1.3077
SE16	7.225749	81.862227		1.3394	0.7633	−0.0830	1.2846
SE17	7.221146	81.862268		1.4223	0.6814	−0.0500	1.3127
SE18	7.21664	81.862094		1.3226	0.7252	−0.0767	1.2405

SE19	7.212376	81.862106	Periya Kalapuwa Deltaic Environment	1.2617	0.6607	−0.1419	1.1040
SE20	7.2072	81.862191		1.4654	0.7606	0.0001	1.3006
SE21	7.204076	81.862158		1.5683	0.8418	0.0242	1.2625
SE22	7.198574	81.862208		1.7626	0.6704	0.1801	1.0638
SE23	7.193826	81.862161		1.5382	0.6423	0.0502	1.3747
SE24	7.18929	81.861817		**2.1443**	0.7908	0.0045	0.9033
SE25	7.184321	81.861851		1.3112	0.7242	−0.0887	1.2417
SE26	7.180015	81.861514		1.5902	0.6853	0.0663	1.2956
SE27	7.176081	81.861185		1.8181	0.6856	0.1572	0.9228
SE28	7.171755	81.861169		1.7712	0.6841	0.1694	1.0488
SE29	7.167286	81.861184		1.3217	0.6254	−0.1257	1.2433
SE30	7.16331	81.861237		1.9831	0.6744	0.0419	0.7387
SE31	7.158451	81.860995		**2.2555**	0.6013	−0.2605	1.0235
SE32	7.153233	81.860743		**2.2512**	0.5905	−0.2680	1.0429
SE33	7.149085	81.860064		**2.1618**	0.6664	−0.2289	0.8571
SE34	7.145328	81.859282		**2.3197**	0.6154	−0.2321	1.2749
SE35	7.140187	81.858415		1.1782	0.8014	0.0708	0.9221
SE36	7.135923	81.857846		1.4564	0.7045	−0.0155	1.3171
SE37	7.131312	81.857338		**2.0696**	0.7688	−0.2124	0.9144
SE38	7.12631	81.857147		1.8138	0.6982	0.1411	0.9447
SE39	7.122209	81.856856		1.8145	0.7654	0.1205	1.0017

SE40	7.118872	81.85863	Korai Deltaic Environment	**2.2252**	0.7683	−0.0514	0.9710
SE41	7.113436	81.858482		1.9763	0.7241	0.1413	0.8218
SE42	7.109029	81.857759		1.6400	0.7342	0.0902	1.2759
SE43	7.104524	81.857756		1.6684	0.7331	0.0980	1.2605
SE44	7.100541	81.857838		1.7437	0.7844	0.0282	0.9797
SE45	7.094531	81.859741		1.6423	0.7289	0.0855	1.2993
SE46	7.09116	81.860833		1.9604	0.6941	0.1497	0.7931
SE47	7.087195	81.861872		**2.1737**	0.6897	−0.1381	0.9074
SE48	7.08273	81.862905		**2.3672**	0.5726	−0.1676	1.2403
SE49	7.078619	81.863356		1.6604	0.9241	−0.1222	0.8788
SE50	7.074585	81.864434		1.2296	0.7624	−0.0772	1.0209

SE51	7.055538	81.868139	Sangamankanda Point	1.6188	0.6824	0.0924	1.3380
SE52	7.052051	81.868409	Coastline	1.6664	0.7619	0.0892	1.2140
SE53	7.048229	81.868891		**2.0170**	0.6797	0.0708	0.7769
SE54	7.042987	81.870117		1.8527	0.7958	−0.0538	0.9059
SE55	7.039525	81.871351		1.2523	0.8803	0.1146	0.8968
SE56	7.035288	81.874681		1.3364	0.8098	−0.0468	1.1138
SE57	7.031769	81.875257		0.9714	0.7054	0.1080	0.7908
SE58	7.026821	81.87818		1.7407	0.7426	0.0714	1.0456
SE59	7.022436	81.878663		**2.2388**	0.6933	−0.1243	1.0444
SE60	7.017852	81.877054		1.0215	0.7035	0.1273	0.8298
SE61	7.014309	81.875717		1.7477	0.6988	0.1491	1.1294
SE62	7.009073	81.873732		1.9366	0.8110	0.0010	0.9320
SE63	7.005745	81.873153		1.8982	0.6843	0.1286	0.7971
SE64	7.001077	81.872357		1.9188	0.6568	0.1035	0.7490

SE65	6.996713	81.870407	Komari Deltaic Environment	1.5277	0.8800	−0.0102	1.0107
SE66	6.992874	81.868241		**2.0663**	0.6548	−0.1162	0.7496
SE67	6.988607	81.86648		1.9659	0.7262	0.0316	0.8226
SE68	6.984367	81.865448		**2.0276**	0.7435	−0.2374	0.8779
SE69	6.979997	81.864945		1.1638	0.7822	−0.0176	0.9358
SE70	6.975583	81.866202		1.6758	0.7477	0.0768	1.1993
SE71	6.97127	81.866788		**2.1646**	0.7171	−0.3708	1.1284
SE72	6.967084	81.865364		1.8333	0.6399	0.2217	0.8252
SE73	6.96373	81.864944		1.9902	0.7395	−0.1794	0.8611
SE74	6.95935	81.863007		**2.2238**	0.7230	−0.1151	1.0282
SE75	6.954769	81.861185		1.8649	0.9079	−0.2543	0.8612

SE76	6.951489	81.859711	Komari Straight Coastline	1.8780	0.8387	−0.1111	0.8815
SE77	6.947508	81.858057		1.7122	0.8429	−0.0564	0.9001
SE78	6.943082	81.856292		**2.0901**	0.6786	−0.2367	0.8263
SE79	6.93864	81.854357		1.7958	0.8043	−0.0106	0.9195
SE80	6.934431	81.852967		1.9747	0.7732	−0.1860	0.8817
SE81	6.928861	81.85183		1.7792	0.7877	0.0537	0.9998
SE82	6.924743	81.850288		**2.0812**	0.6496	−0.1567	0.7609

SE83	6.921036	81.848986	Pottuvil Deltaic Environment	**2.0350**	0.6672	−0.0656	0.7408
SE84	6.916099	81.847131		1.4757	0.7957	−0.0019	1.1878
SE85	6.913073	81.846095		1.7399	0.7380	0.0718	1.0464
SE86	6.907275	81.84428		1.3178	0.7237	−0.0665	1.1769
SE87	6.903729	81.844573		1.1673	0.7714	0.0265	0.9233
SE88	6.899646	81.845048		1.8864	0.7709	−0.1017	0.8947
SE89	6.896997	81.847563		1.3021	0.6267	−0.1484	1.1970
SE90	6.892819	81.846344		**2.0187**	0.7653	−0.1828	0.8697
SE91	6.888113	81.844097		1.3198	0.6792	−0.0994	1.2824
SE92	6.88316	81.843993		1.0825	0.6599	−0.1473	0.7584
SE93	6.878971	81.845085		1.0262	0.7201	0.1017	0.8328
SE94	6.874158	81.845916		1.2732	0.7155	−0.1556	1.2681

SE95	6.87055	81.845088	Arugam Deltaic Environment	1.4019	0.6838	−0.0635	1.3080
SE96	6.867009	81.841852		1.7074	0.7559	0.0931	1.1467
SE97	6.863403	81.838713		1.1464	0.7251	−0.0528	0.9055
SE98	6.860149	81.835619		0.5027	0.6653	0.0075	1.3145
SE99	6.856113	81.833158		0.6480	0.6289	0.1073	1.2694
SE100	6.851778	81.831976		1.9389	0.6805	−0.0200	0.7947
SE101	6.847238	81.831874		1.0236	0.6447	−0.0381	0.7388
SE102	6.843315	81.832284		1.4326	0.6717	−0.0594	1.3311
SE103	6.839528	81.83489		**2.2745**	0.7151	−0.2268	1.4059
SE104	6.838968	81.839646		1.9603	0.6870	0.1130	0.7673
SE105	6.834961	81.839115		**2.5483**	0.5889	0.0272	1.3374

SE106	6.832463	81.835902	Heda Oya Deltaic	1.2075	0.7646	−0.0849	1.0157
SE107	6.828969	81.831941	Environment	1.4497	0.8515	0.0014	1.1497
SE108	6.826926	81.829653		0.9304	1.0179	−0.1192	0.9848
SE109	6.82249	81.825944		0.9354	0.6476	0.0531	0.7412
SE110	6.818324	81.824007		1.7442	0.6563	0.1691	1.0984
SE111	6.813989	81.822672		1.3566	0.7732	−0.0401	1.1251
SE112	6.808836	81.824308		1.1713	0.7832	−0.0419	0.9411
SE113	6.803624	81.824046		1.6203	0.8885	−0.0444	0.8885
SE114	6.80059	81.823339		**2.0815**	0.6487	−0.1863	0.7704
SE115	6.797025	81.822109		1.7395	0.7369	0.0574	1.0509
SE116	6.792697	81.819896		1.9883	0.6959	−0.1471	0.8208
SE117	6.788789	81.81865		**2.1220**	0.6234	−0.2104	0.7965
SE118	6.784332	81.819069		1.3372	0.9281	0.0547	0.8569

SE119	6.76344	81.825404	Wila Oya Deltaic Environment	0.7786	0.9956	0.0218	0.9614
SE120	6.759824	81.822638		1.3176	0.7586	−0.0657	1.1668
SE121	6.756599	81.819133		1.4469	0.8585	−0.0019	1.1157
SE122	6.753752	81.816341		1.3722	0.7617	−0.0404	1.2064
SE123	6.751004	81.813598		1.0656	0.7128	0.0078	0.8319
SE124	6.746975	81.810403		1.4296	0.9645	0.0998	0.7823

The mean grain size of the sediments varies from fine to coarse-grained sand with abundant medium sand (73.38 %). The sorting values are dominated by moderately sorted (55.64 %) to moderately well-sorted (42.74 %). Altogether, 58.87 % of samples showed near-symmetrical skewness. About 25 % of the samples showed coarse-skewed natures while fine-skewed are 16 %. On average, kurtosis values are resulted leptokurtic (35.48 %), mesokurtic (33.06 %) and platykurtic (31.45 %) natures.

**Fig. 3:**
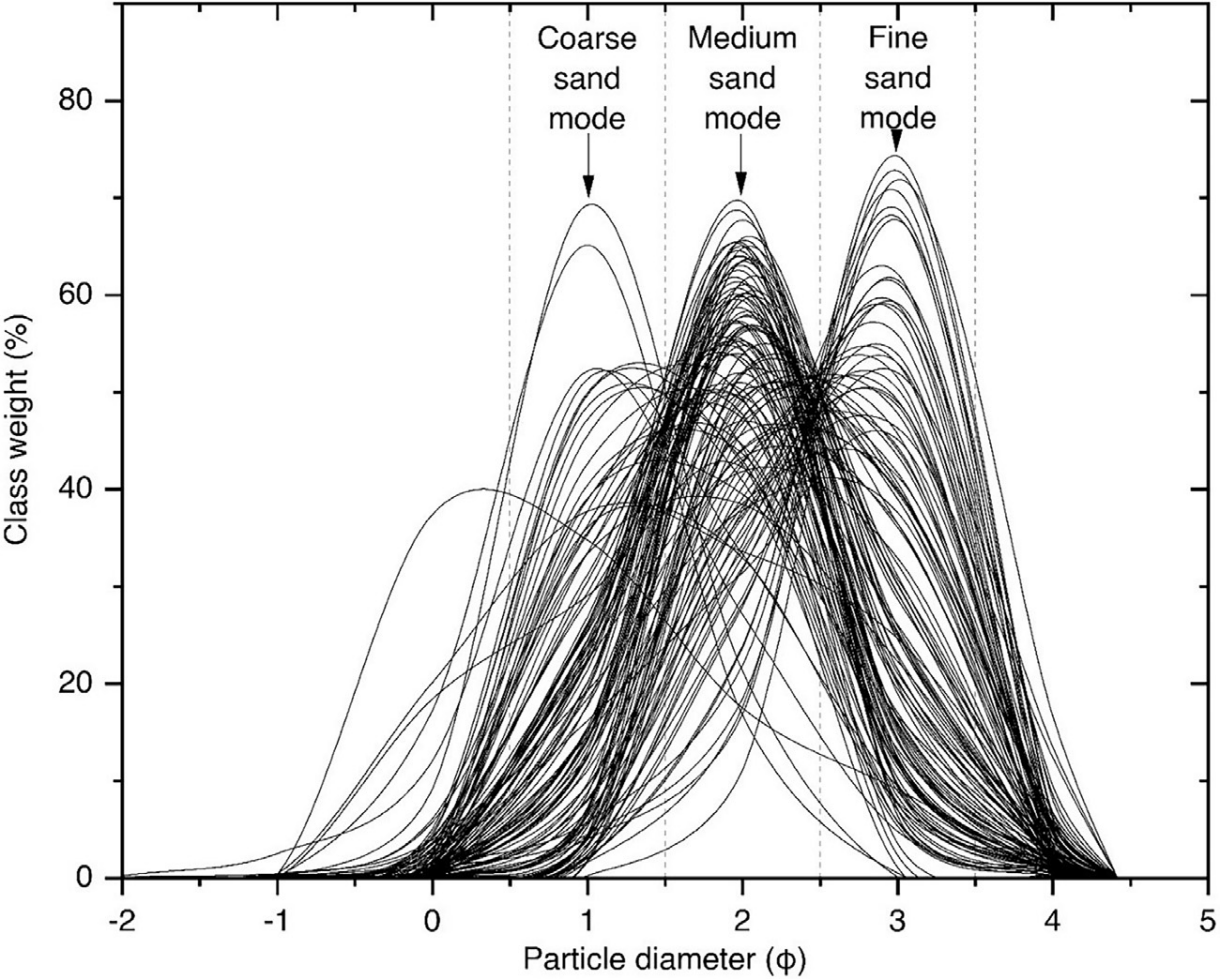
The plot shows the grain size distributions of samples.

In Fig. 3, distributions can be categorized into three modes such as coarse sand, medium sand and fine sand modes. Study samples abundantly show the medium sand populations, while most of the fine sand populations are related to the placers.

**Fig. 4:**
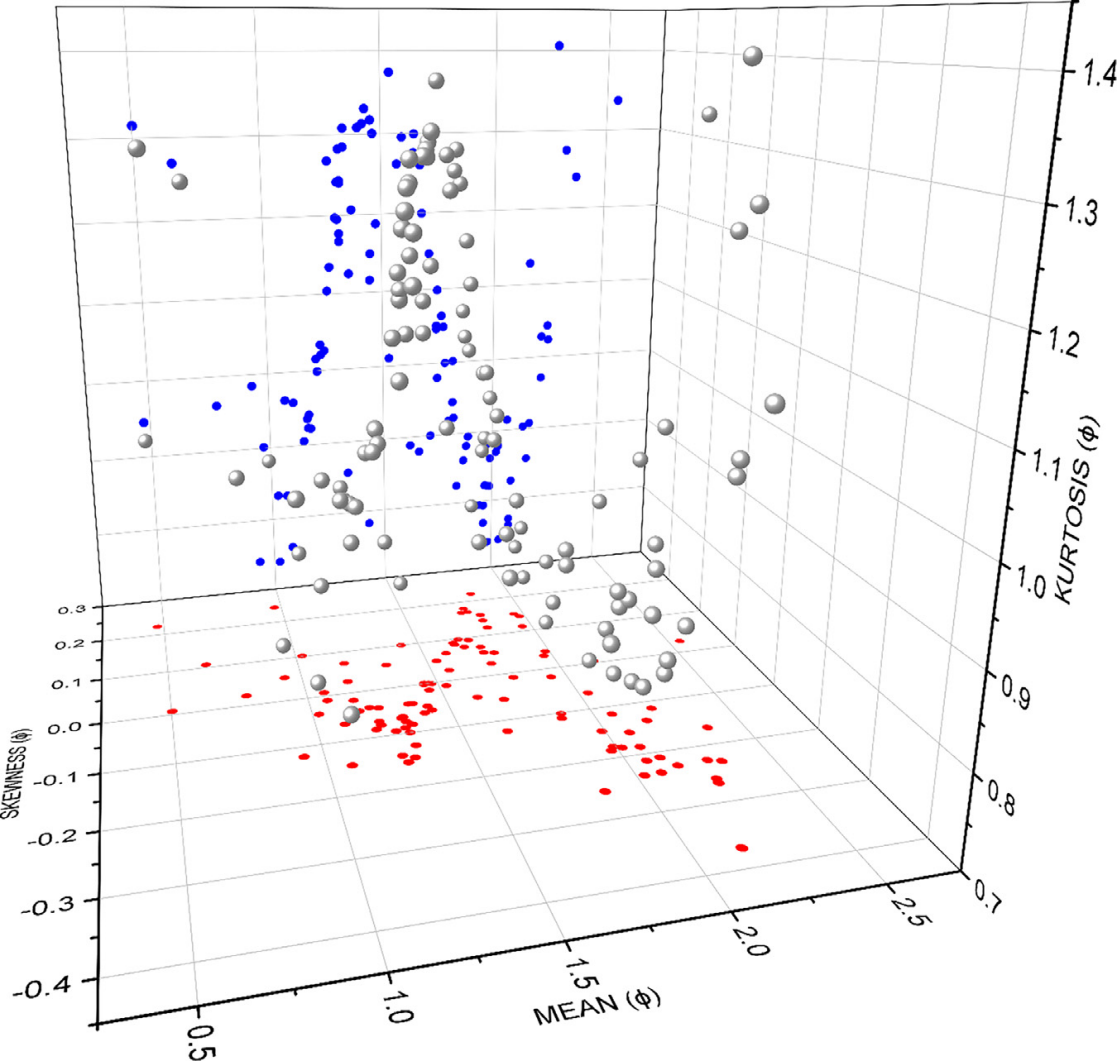
The tri-variate scatter plot shows the variation of mean, skewness and kurtosis of the samples.

The plot shows the sediment population variations from fine, medium to coarse sand through a helical trend. Their bi-variations are projected in the figure in red and blue colours. A similar kind of plot resulted in the study of [5]. As in the mean vs. skewness projection, the studied samples show a zigzag trend that separates four skewed natures. The mean vs. kurtosis projection also shows the clustering of present samples within a sinusoidal trend (Fig. 4).

## 4 Experimental Design, Materials and Methods

Sediment sampling was carried out from October 2019 to February 2020 during the northeast-monsoon season. The sampling season was selected to relays with the higher fluvial outflow time. During the preliminary field visits, placers were recorded between 2 and 3 km near the coastline from fluvial outlets. Hence, the study area was classified into 10 segments based on the fluvial outlets, beach morphologies and depositions types, namely (i) Akkaraipattu Straight Coastline (18 sample points), (ii) Periya Kalapuwa Deltaic Environment (21 sample points), (iii) Korai Deltaic Environment (11 sample points), (iv) Sangamankanda Point Coastline (14 sample points), (v) Komari Deltaic Environment (11 sample points), (vi) Komari Straight Coastline (7 sample points), (vii) Pottuvil Deltaic Environment (12 sample points), (viii) Arugam Deltaic Environment (11 sample points), (ix) Heda Oya Deltaic Environment (13 sample points) and (x) Wila Oya Deltaic Environment (6 sample points). Sampling was designed to find the characteristics of different deposits and coastal types. As a final measure, a total of 124 sediment samples were collected at an interval of 500 m covering a distance of 68 km, from Oluvil to Panama coast (Fig. 1). During sampling, about 5 kg of sediments were collected from a small pit on the swash zone with an average depth of 15–30 cm in each sampling point. Plastic shovels and zip-lock bags were used to collect the samples. Field photographs and sampling locations were recorded at each point.

Before sieve analysis, the samples were air dried to make the grain free from trace moisture. Then, the dried sediment samples were subjected to coning and quartering to obtain representative samples. Carbonate material and salt coatings were removed by 10 % dilute hydrochloric acid and deionized water [6]. These sediment samples were oven-dried at 60 °C for 24 h [6]. Finally, sieve analyses were carried out by Retsch AS 200-digit model digital shaker at 1 phi intervals using ASTM sieves for 15 min [5]. Grain size distribution and graphical statistical parameters namely, mean, sorting, skewness and kurtosis were estimated using the Gradistat Excel template following the [5] method. The tri-bivariate scatter plots of the statistical parameters were plotted using Origin Pro (2021) to understand the relationship between different parameters [5].

## Limitations

Due to the study's budget, the dataset could not capture seasonal variation. Due to coastal defence systems, regular sampling intervals at Akkaraipattu and Panama Beach were disrupted*.*

## Ethics Statement

The current work does not involve human subjects, animal experiments, or any data collected from social media platforms.

## CRediT authorship contribution statement

**Chaturanga Sandaruwan:** Conceptualization, Methodology, Software, Formal analysis, Data curation, Writing – original draft. **Nadeesha Madugalla:** Conceptualization, Validation, Resources, Writing – review & editing, Supervision. **Madurya Adikaram:** Conceptualization, Validation, Resources, Writing – review & editing, Supervision. **Amarasooriya Pitawala:** Conceptualization, Resources, Writing – review & editing, Supervision.

## Data Availability

Grain-size distribution dataset of lagoonal and riverine coastal placer deposits along the southeastern coast of Sri Lanka (Original data) (Mendeley Data).
